# Plant-derived extracellular vesicles quality control: key process progress and future research directions​

**DOI:** 10.1186/s11671-025-04399-0

**Published:** 2025-12-16

**Authors:** Fuxing Shu, Yuhe Hu, Surendra Sarsaiya, Leilei Jin, Xue Yang, Fengjian Liu, Jianguo Zhu, Guoguang Chen, Jishuang Chen

**Affiliations:** 1 School of Biotechnology and Pharmaceutical Engineering, Nanjing Tech University, Nanjing, Jiangsu China; 2 Bioresource Institute for Healthy Utilization, Zunyi Medical University, Zunyi, Guizhou China; 3 Jianguo Zhu Department of Urology, Guizhou Provincial People’s Hospital, Guiyang, 550002 Guizhou China; 4 The Medical College of Guizhou University, Guiyang, 550002 Guizhou China

**Keywords:** Raw material, Isolation and purification, Characterization, Storage, Drug delivery systems, Biomedical applications, Process optimization

## Abstract

Plant-derived extracellular vesicles (PDEVs) have garnered significant attention as crucial mediators of intercellular and cross-species communication in plants, owing to their unique bioactivity and medicinal potential. However, quality control of PDEVs is of paramount importance for their functionality and applications. This review systematically summarizes the key aspects that influence PDEVs quality, including source material preparation, isolation and purification, characterization, and storage conditions. Regarding source material preparation, the genetic background, abiotic factors (such as light, temperature, and moisture), biotic factors (including pathogen infection and probiotic effects), and traditional processing methods significantly affect the composition and function of PDEVs. Plant tissue culture techniques and temporary immersion bioreactor systems (TIBS) have substantial potential for addressing the issue of fluctuating raw material quality. During the isolation and purification processes, existing methods (e.g., ultracentrifugation, polymer precipitation, and size-exclusion chromatography) have their respective advantages and disadvantages, necessitating the optimization of operations based on specific requirements to avoid vesicle damage or impurity introduction. In terms of characterisation, integrating techniques such as transmission electron microscopy (TEM), nanoparticle tracking analysis (NTA), and western blotting can comprehensively evaluate the quality of PDEVs. Additionally, storage conditions significantly affect the stability of PDEVs, and appropriate measures should be adopted to reduce degradation. Future research should focus on developing standardized preparation processes and efficient characterization techniques to facilitate the widespread application of PDEVs in drug delivery, functional foods, and other fields.

**Graphical abstract:**

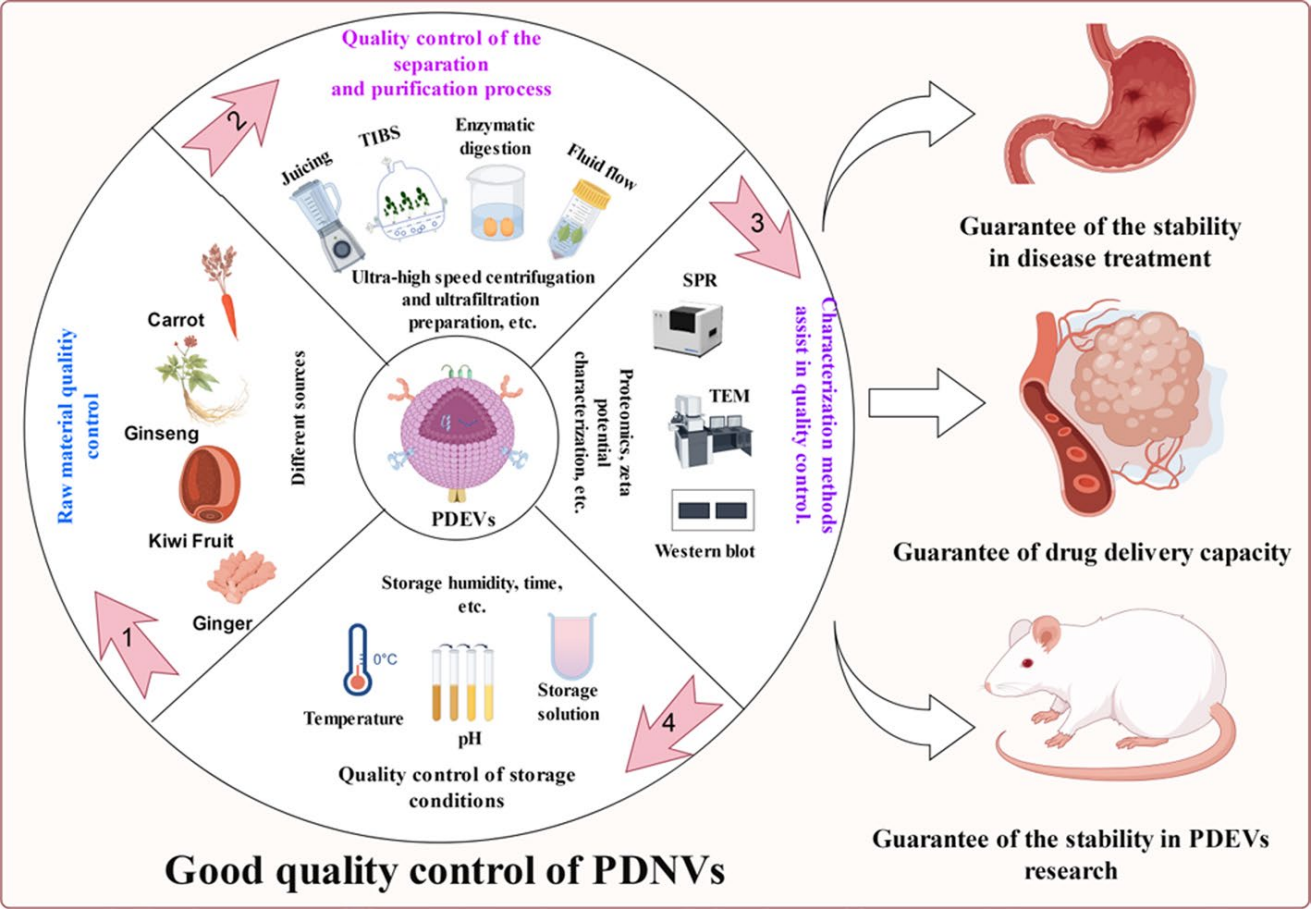

## Introduction

Plant-derived extracellular vesicles (PDEVs) are nanoscale membrane-bound vesicles released by plant cells into the extracellular space, and are characterized by a lipid bilayer structure [1]. Although they share similarities with mammalian extracellular vesicles (EVs), they are distinct because of their plant origin and specific metabolites [2]. PDEVs are typically within the nanometer range, often between 30 and 150 nm, and are enriched with lipids, proteins, nucleic acids (such as small RNAs), and pharmacologically active compounds. Therefore, they serve as essential mediators of material exchange and information transfer between plant cells and across species [3].

Virtually all plant cells, including those from tissues such as roots, stems, leaves, and fruits, secrete extracellular vesicles [4]. These vesicles predominantly form through the endosomal system or multivesicular body (MVBs) pathway, ultimately fusing with the plasma membrane for extracellular release [5]. Some PDEVs may arise directly from plasma membrane budding [6]. To date, PDEVs have been isolated from a diverse range of plants, including medicinal and edible plant varieties. Notably, the composition and function of these vesicles vary significantly across plant species [3, 7–9].

The structure and composition of PDEVs can be categorized as follows: (1) Membrane structure: Encased by a lipid bilayer, PDEVs resemble mammalian extracellular vesicles in structure. However, plant-specific lipids (such as phytosterols) and membrane proteins may confer unique stability to PDEVs [10, 11]. (2) Nucleic acids: PDEVs carry small RNAs, mRNAs, and other nucleic acids that potentially participate in the regulation of cross-species gene expression regulation [5, 12]. (3) Proteins: These include enzymes, signalling proteins, and plant-specific proteins [13]. (4) Lipids: Rich in phospholipids and sphingolipids, may be associated with the biocompatibility and targeting ability of vesicles [10]. (5) Secondary metabolites: Bioactive molecules such as flavonoids and terpenoids contribute to the pharmacological effects of PDEVs [14, 15]. Additionally, PDEVs generally exhibit a uniform particle size distribution, whereas their surface charge (zeta potential) influences both stability and cellular uptake efficiency [16].In terms of physical properties, the particle size of most PDEVs is concentrated in the range of 30–150 nm. This range ensures the penetration of target tissues and prolongs in vivo circulation time through the "enhanced permeability and retention (EPR) effect," thereby avoiding rapid clearance [17]. The zeta potential of PDEVs is mostly a negative value ranging from − 10 to − 40 mV, which maintains colloidal stability through charge repulsion and balances cellular uptake efficiency^[18]^. Additionally, structural stability directly affects the encapsulation efficiency and leakage of active ingredients.In terms of chemical properties, the membrane of PDEVs is centered on a lipid bilayer and contains components such as phospholipids and phytosterols, which enhance biocompatibility and targeting specificity [9]. The exposed functional groups on the surface enable the optimization of targeting ability and circulation time through chemical modification. Furthermore, the weakly acidic internal lumen environment, with a pH of 5.0–6.5, and the antioxidant components, such as vitamin C and glutathione, within PDEVs can stabilize active ingredients [19].

The objective of this review is to systematically analyze the current understanding of PDEVs, focusing on the critical challenges and advancements in quality control processes, including source material preparation, isolation and purification techniques, characterization methods, and storage conditions, to ensure their functional integrity and therapeutic efficacy. By evaluating their biological roles in plants, pharmaceutical potential as nanodrug delivery systems, and applications in biomedical fields such as anti-inflammatory, anticancer, and regenerative therapies, this review aims to highlight existing gaps in standardization and scalability while proposing future research directions, such as integrating synthetic biology, artificial intelligence, and multidisciplinary innovations, to optimize PDEVs for clinical translation and sustainable industrial utilization.

## Plant-derived extracellular vesicles: bridging biological roles and therapeutic innovations

In plants, research on the functions of PDEVs remains limited, but accumulating evidence confirms their critical regulatory roles—all dependent on the structural integrity and cargo accuracy guaranteed by quality control. PDEVs are core mediators of systemic acquired resistance (SAR): upon pathogen infection, they deliver regulatory RNAs to target cells to effectively inhibit pathogenic gene expression [20], carry antibacterial proteins or metabolites that directly disrupt the cellular structures of bacteria and fungi [21], and even interact with pathogen-derived vesicles to neutralize their toxicity [12], thereby activating intercellular immune signal transmission [22]. They also serve as long-distance transport carriers for nucleic acids and proteins to coordinate inter-tissue physiological responses, such as extracellular vesicles released from roots may carry stress response factors to regulate the stress resistance of aboveground plant parts [23, 24]. Additionally, PDEVs participate in plant development, maintaining seed viability by transferring lipids and RNAs that influence seed germination and seedling growth [25]; without quality control to avoid structural damage or cargo loss, these physiological functions would be severely impaired. (Table 1).

**Table 1:** Therapeutic effects of plant-derived extracellular vesicles (PDEVs) in preclinical disease models

Plant Source		Disease Model	Therapeutic Effects	References
	Cannabis (*Cannabis sativa*)	Pigmentation disorders	Modulating the ERK/Akt axis during excessive melanin synthesis	[20]
	Java Brucea(*Brucea javanica*)	Cancer	Suppresses tumor growth, metastasis, and angiogenesis in breast cancer mouse models with high biosafety	[21]
	Cactus fruit(*Opuntia ficus-indica*)	Wound healing	Accelerates normal wound healing by modulating cellular responses	[12]
	Aloe(*Aloe vera*)	Wound healing	Reduces inflammation, inhibits myofibroblast differentiation/contraction, and accelerates healing	[22]
	Black Nightshade(*Solanum nigrum*)	Inflammation	Reduces *IL-6* gene and protein expression by up to 97.28%	[23]
	Basil(*Ocimum basilicum*)	Cancer	Exhibits potent anticancer effects against pancreatic cancer cells	[24]
	Dandelion(*Taraxacum officinale*)	Wound healing	3D structure promotes cell proliferation/migration and accelerates healing of infected wounds with TH-EVN cooperation	[25]
	Maca(*Lepidium meyenii*)	Depression	Enhances 5-HT release via gut-brain axis modulation to alleviate depressive behaviors	[26]
	Notoginseng (*Panax notoginseng*)	Ischemic stroke	Lipid components attenuate cerebral ischemia/reperfusion (CI/R) injury by shifting microglial phenotype from pro-inflammatory M1 to anti-inflammatory M2 via pI3k/Akt pathway	[27]
	Tomato(*Solanum lycopersicum*)	Gut microbiota dysbiosis	Reverses Fusobacterium nucleatum-induced dysbiosis in gut microbiota	[28]
	Blueberry(*Vaccinium corymbosum*)	Cytotoxicity	Absorbed by Caco-2 cells without cytotoxicity	[29]
	Edelweiss(*Leontopodium nivale*)	Skin whitening	Reduces melanin production	[30]
	Purslane(*Portulaca oleracea*)	Colitis	Alleviates DSS-induced colitis by promoting CD4 + CD8 + T-cell expansion	[31]
	Madagascar Periwinkle(*Catharanthus roseus*)	Immune enhancement	Boosts immunity via TNF-α/NF-κB/PU.1 axis	[32]
	Yam (* Dioscorea spp.*)	Osteoporosis	Yam-derived exosome-like nanovesicles stimulate osteoblast formation and prevent osteoporosis in mice	[33]
	Broccoli(*Brassica oleracea var. Italica*)	Cancer	Selenium-enriched broccoli EVs inhibit pancreatic adenocarcinoma; suppress Caco-2 activity (50–70%) and HepG2 cell viability (50%)	[34]
	Lemon(*Citrus limon*)	Cancer	The novel nanoplatform (LEVBD) is formed by embedding the membrane fragments from breast cancer cell with the lemon-derived nanovesicles (LEVs) as the foundational skeleton	[35]
	Ginseng(*Panax ginseng*)	Cancer	Facilitate DCs maturation through TLR4	[36]
	Moringa(*Moringa oleifera*)	Cancer	Selectively cytotoxic to Jurkat and HeLa cells	[37]
	Tea plant flower(*Camellia sinensis*)	Cancer	Strong cytotoxicity against breast cancer cells; inhibits lung metastasis in vivo	[38]
	Cauliflower(*Brassica oleracea var. Botrytis*)	Inflammation	Protects RAW264.7 cells from LPS-induced inflammation by suppressing pro-inflammatory cytokines	[39]
	Garlic ( *Allium sativum*)	Inflammation	Protects HepG2 cells from LPS-induced inflammation and alleviates DSS-induced colitis	[40]
	Strawberry(*Fragaria* × *ananassa*)	Antioxidant	Protects adipose-derived mesenchymal stem cells from oxidative stress	[41]
	Blueberry(*Vaccinium corymbosum*)	Antioxidant	Protects EA.hy926 cells from TNF-α-induced cytotoxicity and oxidative stress	[42]
	Carrot(*Daucus carota subsp. Sativus*)	Anti-inflammatory/Antioxidant	Protects H9C2 cells from oxidative stress and inhibits Nrf-2 downregulation	[43]
	Ginger(*Zingiber officinale*)	Inflammation	Suppresses DSS-induced colitis progression	[44]
	Grape(*Vitis vinifera*)	Anti-inflammatory	Enhances Nrf-2 nuclear translocation in RAW264.7 cells	[45]
	Grapefruit(*Citrus* × *paradisi*)	Autoimmune diseases/Inflammation	Treats psoriasis and atopic dermatitis; protects against DSS-induced colitis by inhibiting pro-inflammatory cytokines and colonic macrophages	[45]
	Tea(*Camellia sinensis*)	Inflammation	Delays mammary tumor growth via apoptosis and microbiota modulation; preferentially uptaken by macrophages to upregulate *HO-1* and *IL-10*	[46]

In biomedicine, PDEVs show significant potential as next-generation bio-derived nanodrug delivery systems, and their application value can only be realized through rigorous quality control. Compared to synthetic nanoparticles, such as liposomes with unresolved toxicity and high production costs [46, 47], and mammalian-derived EVs, PDEVs possess unique advantages: They are primarily isolated from edible plants, with their non-toxicity verified by in vivo and in vitro studies [48], PDEVs possess gastrointestinal stability and inherent targeting ability, and can act synergistically with loaded drugs.Engineering modifications further expand the applications of PDEVs—such as surface modifications, for example, displaying a GalNAc ligand [49] or modifying membrane proteins with a HaloTag, which [50] enhance targeting. These modifications enable them to deliver antitumor drugs, antifibrotic drugs, or cross the blood–brain barrier. However, challenges such as non-uniform isolation techniques, low yield/purity [51, 52], low exogenous drug-loading efficiency that can damage PDEV integrity [53], and inherent heterogeneity still exist. Addressing these issues relies on optimizing quality control. Notably, quality control is the core prerequisite for realizing the value of PDEVs, whether in supporting plant physiological processes or promoting biomedical translation. It ensures stable structural integrity, consistent compositional characteristics, and reliable functional activity, thereby bridging their basic biological roles with practical applications.

## Quality control of PDEVs: challenges, strategies, and implications for biomedical applications

Current research on PDEVs has predominantly focused on their roles within plants and cross-kingdom regulatory functions, relegating quality control to a peripheral role. However, quality control of PDEVs is a critical factor in determining their efficacy. Without stringent quality control, even the most promising post-preparation research and extensive application may have unpredictable consequences. Quality control of PDEVs can be categorized into four key aspects: source material preparation, isolation and purification, characterization, and storage. Among these, source material preparation is extremely important, as it dictates nearly all aspects of vesicle composition. Nevertheless, each process is integral to the quality control system and uniquely contributes to the overall process.

### Influencing factors of raw material quality control and current mitigation strategies

#### Impact of genetic background of raw materials on PDEVs quality control

The genetic background of plants influences the composition of PDEVs at two levels: interspecies differences and intraspecies variations, both of which directly determine the types of bioactive substances carried by vesicles and their functional properties. The genetic makeup of different plant species shapes their metabolic pathways and synthesis of secondary metabolites, resulting in significant variations in PDEVs composition. For example, citrus-derived PDEVs (CEVs) are enriched with specific miRNAs that may contribute to antioxidant and anti-inflammatory activities [54]. Conversely, PDEVs from medicinal plants such as Cannabis may carry small RNAs associated with immunomodulation [7]. PDEVs from cruciferous plants, such as radishes, contain unique antiproliferative proteins that potentially inhibit the tumor cell cycle [55], whereas those from grapes and berries are rich in polyphenols, which are correlated with their antioxidant capacity [56]. PDEVs from plants grown in organic agriculture exhibit higher concentrations of antioxidant components, highlighting the interactive effects of genetic background and environment on their composition [56]. These differences can be attributed to plant species-specific gene expression profiles that regulate the selective packaging of substances during vesicle formation, such as variations in transporter and RNA-binding proteins [10, 57].

Even within the same species, genetic variations among subspecies and cultivars can lead to compositional changes in the PDEVs. For example, significant differences in flavonoid content exist among CEVs from different citrus cultivars (such as sweet orange and lemon), which correlates with the activity of cultivar-specific metabolic enzymes [11, 58]. In different cultivated subspecies of medicinal plants such as Ginseng, PDEVs may carry varying proportions of ginsenosides, affecting their antitumour or immunomodulatory effects [9]. The miRNA composition of extracellular vesicles from different Arabidopsis ecotypes differs, potentially influencing cross-species gene silencing efficiency. Modifications can alter the expression of key metabolic enzymes (such as P450 enzymes), thereby affecting vesicle composition [12, 59], Single-nucleotide polymorphisms (SNPs) and epigenetic modifications [7, 15]. The genetic background of plants significantly influences the nucleic acid, protein, lipid, and secondary metabolite composition of PDEVs by regulating metabolic pathways and the mechanisms of substance packaging. This characteristic provides a basis for precise screening of functionally specific vesicles. For example, plant sources can be optimized by breeding or gene editing to obtain PDEVs for specific therapeutic applications [5, 60].Future research should integrate multi-omics technologies (such as transcriptomics and metabolomics) to elucidate the relationship between genetic markers and vesicle composition.

#### Impact of abiotic factors on PDEVs quality control via raw materials

As crucial signalling molecules in plants, PDEVs respond to abiotic environmental stimuli, and their composition and function undergo alterations that enable plants to adapt to these changes. The specific impacts include diverse environmental factors such as light conditions, temperature stress, humidity and water conditions, and soil factors. In terms of light conditions, light of different wavelengths, such as ultraviolet (UV), blue, and red, can modulate plant cell metabolism, thereby altering the content of bioactive molecules in PDEVs. For instance, UV light may induce plants to produce more antioxidant substances like phenolics and flavonoids, which are encapsulated within PDEVs to enhance their antioxidant capacity [56], while red or blue light affects the abundance of energy metabolism-related proteins (e.g., ATPases) or nucleic acids (e.g., miRNAs) in PDEVs by influencing photosynthetic efficiency [57], and photoperiod, specifically changes in circadian rhythms, may regulate the secretion frequency or dynamic composition of PDEVs [61]. Temperature stress also plays a significant role, as extreme temperatures trigger stress responses in plant cells, leading to the enrichment of stress-related proteins (such as heat shock proteins (HSPs) and stress-responsive small RNAs (e.g., miR398) in PDEVs [57, 62]), whereas low temperatures may increase the proportion of unsaturated fatty acids in PDEVs to maintain their membrane fluidity [63], which affects the stability of plant cell membranes, thereby altering the secretion amount or particle size distribution of PDEVs [64]. Humidity and water conditions are equally critical: water is essential for plant survival, and the plant's response to water stimuli has been a key aspect throughout its evolution over 3.5 billion years, such that under water-deficient conditions, plants may transmit more drought-resistant signalling molecules (such as metabolites or small RNAs involved in the abscisic acid (ABA) pathway through PDEVs to coordinate intercellular defense responses [61, 62], which increases the risk of pathogen infection, prompting plants to secrete PDEVs rich in antibacterial proteins (such as chitinases) and defensive miRNAs [65, 66]. Lastly, soil factors cannot be overlooked, as soil provides plants with nutrients and physical support, and the complex nutrient elements in soil endow medicinal herbs in specific regions with unique efficacy, as exemplified by the concept of "authentic medicinal materials" in traditional Chinese medicine (TCM), with these soil nutrients significantly influencing the composition and function of PDEVs: for example, nitrogen or phosphorus deficiency may prompt PDEVs to carry more nutrient transporter proteins or miRNAs (such as miR167) that regulate root development and optimize resource acquisition [57, 67], plants exposed to heavy metal pollution (such as cadmium and lead) may excrete toxic substances or transmit detoxification-related proteins (such as metallothioneins) through PDEVs [62], and air pollutants (such as ozone and polycyclic aromatic hydrocarbons (PAHs)) can increase the levels of oxidative stress-related components (such as superoxide dismutase and glutathione) in PDEVs, alleviating cellular damage [62, 68].

#### Impact of biotic factors on PDEVs quality control via raw materials

The composition of plant-derived extracellular vesicles (PDEVs) is dynamically regulated by various biotic factors including pathogenic microorganisms, probiotics, viruses, and herbivores. These factors significantly influence the type and abundance of proteins, nucleic acids, lipids, and secondary metabolites carried by PDEVs through direct or indirect molecular interactions.

Upon infection by pathogenic microorganisms such as bacteria and fungi, plants undergo stress responses triggered by the secretion of effector proteins or toxins, leading to alterations in the composition [69]. The RNA or DNA fragments of pathogens may be taken up by plant cells and incorporated into PDEVs, establishing a cross-species nucleic acid transfer mechanism in plants. For example, in response to pathogen infection, miRNAs carried by PDEVs may target pathogen genes to inhibit their proliferation [70].

Probiotics such as plant growth-promoting rhizobacteria affect plant metabolism through symbiotic relationships, thereby regulating the composition of PDEVs. Metabolites secreted by probiotics, including short-chain fatty acids and lipopeptides, can activate plant cell signalling pathways (e.g., the jasmonic acid pathway), inducing the enrichment of anti-inflammatory or immunomodulatory molecules (such as antioxidant enzymes and phenolic compounds) in PDEVs [71]. Additionally, upon contact with plant roots, extracellular vesicles secreted by probiotics may carry symbiosis-related proteins (e.g., nodulation factors), facilitating cross-species signal transduction via PDEVs and promoting the transport of mRNAs related to plant auxin synthesis [66].

To defend against viral infections, PDEVs may accumulate small antiviral molecules (such as saponins and alkaloids) and defense-related miRNAs (e.g., the miR168 family that targets viral replicases) [54, 72]. When herbivores consume plant tissues, mechanical damage or saliva secretion triggers the secretion of defensive components in the plant PDEVs. The physical damage caused by herbivory activates the jasmonic acid (JA) and salicylic acid (SA) signalling pathways in plants, inducing the enrichment of defense proteins such as protease inhibitors and polyphenol oxidases in PDEVs to mitigate further tissue consumption [73, 74].

Beyond the aforementioned factors, microbial communities (such as soil symbiotic flora) and competing plants can indirectly affect the PDEVs composition through the secretion of quorum-sensing molecules or allelochemicals. For example, allelochemicals released by neighboring plants may transmit competitive signals via root-derived PDEVs, thereby altering the secondary metabolite profiles of the recipient plant vesicles [57, 75].

**Fig. 1:**
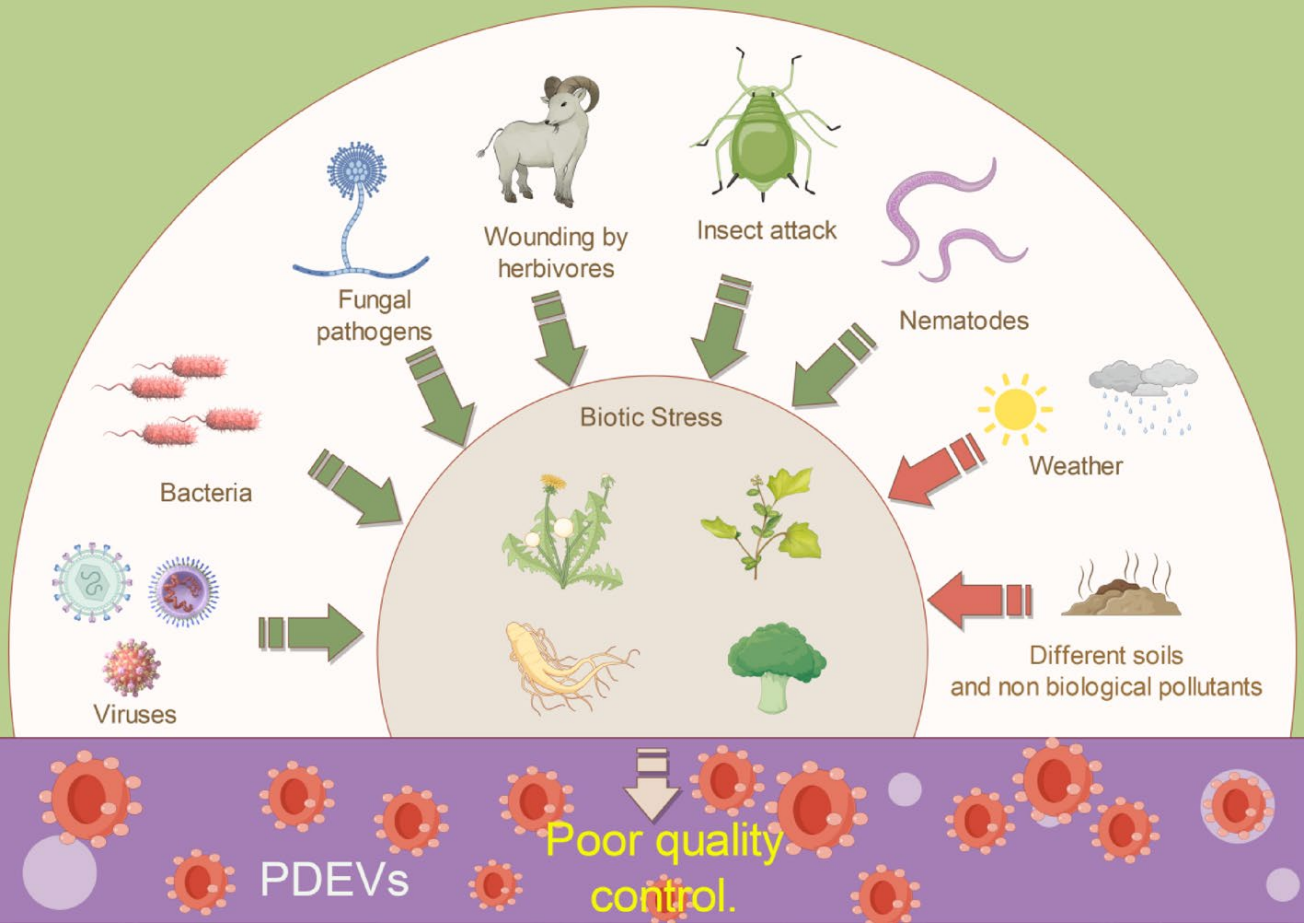
Illustration of the uncontrollable quality of PDEVs in plants cultivated under natural environments due to biotic or abiotic factors

In conclusion, biotic factors exhibit high specificity for regulating PDEVs composition, with mechanisms involving gene expression reprogramming, metabolic pathway activation, and cross-species molecular exchange. These dynamic changes render PDEVs crucial mediators for plant adaptation to complex biotic environments (Fig. 1).

#### Impact of processing of chinese herbal medicines on PDEVs quality control

Traditional processing methods for Chinese herbal medicines, including stir-frying and steaming, involve high temperatures, acid–base treatments, and other physical and chemical manipulations. These processes may directly disrupt the lipid bilayer structure of extracellular vesicles, leading to leakage of their contents and loss of biological activity. The inherent fragility of extracellular vesicles makes them prone to membrane rupture under high temperatures or strong oxidative conditions, thereby compromising their function in intercellular signal transmission [76]. For example, although ultrasonic-assisted extraction can enhance extraction efficiency, excessive treatment may damage the integrity of extracellular vesicles [77, 78]. Alcohol- or vinegar-based processing methods commonly used in traditional Chinese medicine may alter the surface charge of extracellular vesicles, affecting their binding ability to target cells [79]. The activity of extracellular vesicles depends not only on their physical structure but also on the bioactive molecules they carry, such as plant-specific miRNAs [80]. Enzymatic hydrolysis or oxidation reactions during processing may degrade small RNAs within extracellular vesicles, weakening their ability to regulate gene expression in recipient cells. For instance, the content of gingerol-related metabolites in ginger-derived extracellular vesicles (GDEVs) decreases significantly after high-temperature drying, resulting in a decline in their antitumour activity [81].

Extracellular vesicles are dynamic active carriers, and their functions rely on the coordinated action of an intact membrane structure and the contained substances, which differs from the stability-dependent activity of common chemical components, such as flavonoids and alkaloids. The activity of extracellular vesicles is closely related to the conformation of their membrane proteins, including HSPs and transporters [78]. However, thermal denaturation or chemical modification during processing may inactivate these proteins, blocking their targeted delivery functions [76, 79]. Traditional decoction methods may damage adhesion molecules on the surface of extracellular vesicles, preventing them from recognizing diseased cells [82].

#### Current measures for raw material quality control

As discussed above, traditional processing methods for Chinese herbal medicines are detrimental to plant-derived extracellular vesicles (PDEVs) because PDEVs exhibit optimal activity in their fresh state. Therefore, fresh and unprocessed plants are preferred for the extraction of PDEVs. However, uncontrolled biotic and abiotic factors in the natural environment significantly affect PDEVs. Despite the emergence of standardized cultivation [83] and wild-simulated cultivation [84] in recent years, these factors remain challenging to control. Plant tissue culture technology provides a sterile environment that protects the plants from microbial interference. By cultivating plants under identical genetic backgrounds with consistent media, lighting, humidity, and temperature conditions, this technology maximally mitigates the impact of abiotic factors, thereby enabling the production of high-quality PDEVs. In addition to enabling large-scale, year-round production, plant tissue culture technology allows for targeted regulation of metabolite production. Consequently, this technology has shown great potential in the research and application of PDEVs, and has received increasing attention in recent years [32].

### Impact of isolation and purification on PDEVs quality control

The isolation and purification of PDEVs involve two major steps: raw material treatment and purification, both of which are crucial for ensuring the sustainability and purity of the vesicles. This process ranks second only to raw-material cultivation in terms of the importance of PDEVs quality control.

#### Raw material treatment

Currently, there are three primary methods of isolating PDEVs. The first method involves extracting the entire plant or specific plant parts to produce "juice" [85]. However, this approach may yield artificial vesicles and sacrifice plants, preventing the continuous acquisition of PDEVs from plants with the same genetic background. The second method utilizes cellulase to digest the cell wall, followed by the termination of the enzymatic reaction [51]. This method not only damages the plant, but also introduces impurities from digestive enzymes. Prolonged digestion can harm the vesicles, and the method is cumbersome and costly, resulting in low yields, making it less than ideal for use. The third method focuses on isolating extracellular vesicles (EVs) from apoplastic fluid derived from leaves [86] or seeds [87]. Although this method can collect genuine PDEVs, it suffers from low yields and is largely limited to the leaf or seed tissues.

**Fig. 2:**
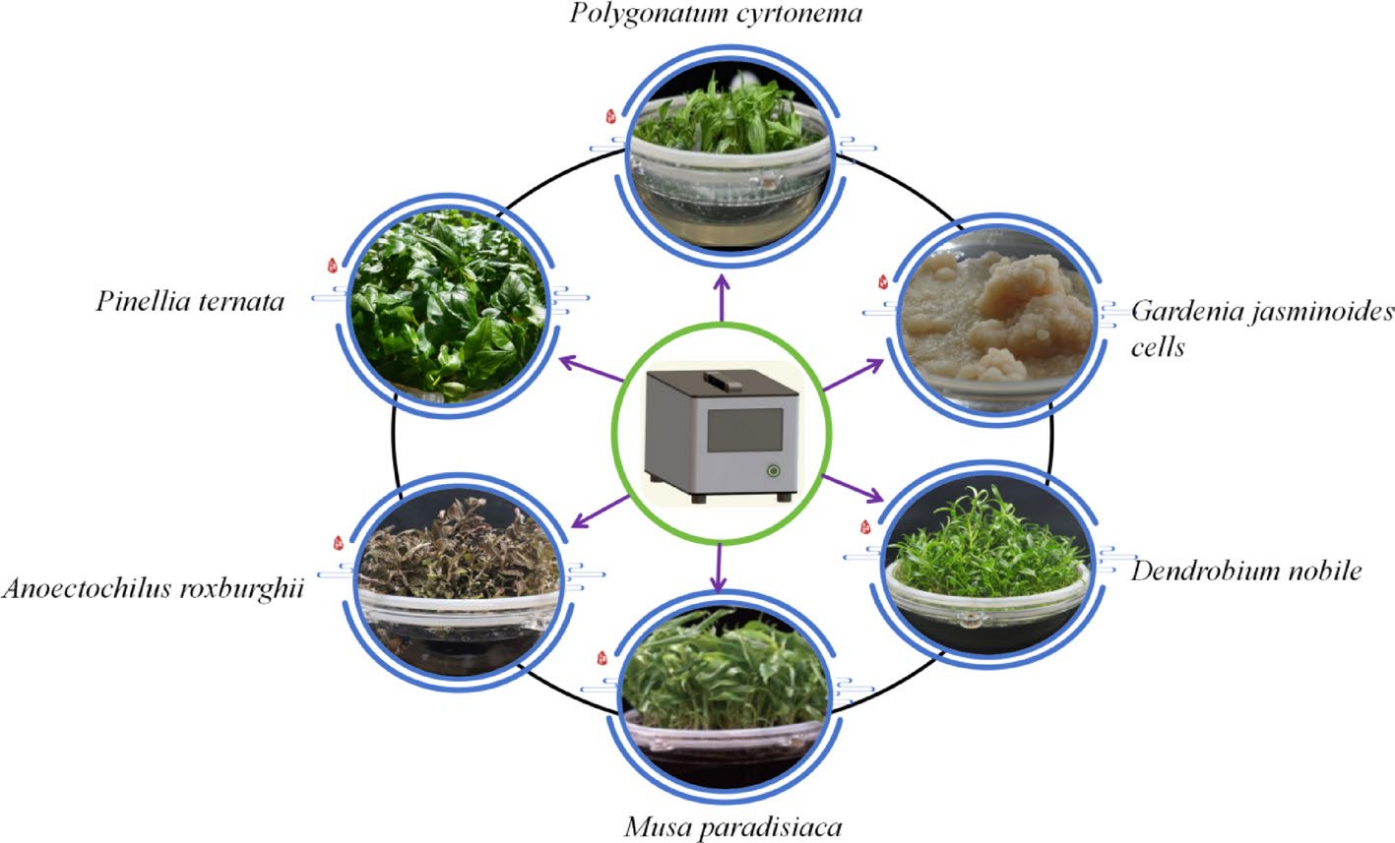
Cultivation of raw materials for some PDEVs was prepared by TIBS

An ideal scenario for the isolation and purification process is to achieve high-quality, continuous production of pure PDEVs in an economical manner without damaging the plants (i.e., maintaining a consistent genetic background). Evidently, these three methods do not satisfy these criteria. As previously mentioned, plant tissue culture technology has significant potential for quality control of PDEVs. Plant tissue can be cultured using solid, liquid, and temporary immersion culture methods. Solid culture can control plant quality, but obtaining PDEVs requires icing, enzymatic digestion, cytoplasmic streaming, etc., which is one of the main methods that can ensure a certain level of PDEVs quality control, but is not a perfect solution. Liquid culture is favorable because plants release their extracellular vesicles into the culture medium during cultivation. PDEVs can be collected from the medium without damaging plants, and continuous production of pure PDEVs from plants with the same genetic background can be achieved by simply changing the culture medium. However, a significant drawback of this method is that prolonged immersion can cause vitrification during plant growth, potentially leading to abnormal PDEVs. Temporary immersion culture provides plants with controlled immersion time and frequency according to their needs, thereby accelerating their growth and preventing vitrification. This method demonstrated nearly ideal results for PDEVs quality control (Fig. 2). Based on the principle of temporary immersion, a temporary immersion bioreactor system (TIBS) has been developed, which has shown remarkable capabilities in the cultivation of *Pinellia ternata* [88]and the quality control of its extracellular vesicles [3, 89].

#### Impact of purification on PDEVs quality control

Current purification methods for extracellular vesicles primarily include ultracentrifugation, sucrose density gradient centrifugation, polymer precipitation (e.g., polyethylene glycol precipitation), size-exclusion chromatography (SEC), immunoadhesion purification (e.g., using L1CAM antibody), ion-exchange chromatography, and microfluidic/acoustic-assisted technologies. Each technique has its advantages and disadvantages (Table 2). In the context of quality control, procedures that are prone to cause vesicle rupture or introduce impurities, such as those involving capture antibodies, should be avoided or optimized.

**Table 2:** Advantages and Disadvantages of Existing Purification Methods for PDEVs

Purification Method	Principle	Advantages	Disadvantages	Application Examples
Ultracentrifugation (UC)	Separation of EVs based on differences in density and sedimentation velocity	Traditional gold standard; no additional reagents required; suitable for large-scale samples [90, 91]	low recovery rate (especially for small EVs)[92, 93];	This method rapidly concentrated EVs from large citrus juice samples to enrich them for subsequent proteomic or RNA-seq analysis [110]
Polymer Precipitation Method (e.g., PEG)	Precipitation of EVs by reducing their solubility with polymers	Simple operation, low cost; suitable for clinical rapid testing[94, 95]	Low purity (easily co-precipitates non-EV impurities)[92, 96]; may introduce polymer interference in downstream analysis[95]	Grapefruit EVs isolated by this method served as nanocarriers for doxorubicin, enhancing drug targeting and antitumor activity[96]
Size Exclusion Chromatography (SEC)	Separation of EVs by particle size, retaining biological activity	High purity (reduces protein contamination); suitable for functional research[97, 98]	Low flux, time-consuming[98]; difficult to distinguish EV subgroups (such as exosomes and microvesicles)[99, 100]	SEC isolated high-purity EVs from broccoli sprouts, enabling the study of their chemopreventive cargo and mechanisms of disease prevention[100]
Immunoprecipitation (e.g., L1CAM)	Capture of target EVs using antibodies specific to certain surface markers	High specificity; suitable for disease marker research[101, 102]	High cost, suitable only for specific EV subgroups [102]; may miss EVs without markers [103]	Anti-PAT1 magnetic beads specifically isolated distinct Brassicaceae-specific EVs subpopulations from mixed plant saps [102]
Ion Exchange Chromatography	Separation of EVs based on differences in surface charge (e.g., anion exchange)	Scalable; maintains EV integrity [90, 104]	Requires optimization of buffer conditions to avoid EV damage [105]; limited effectiveness for complex samples [106]	Method isolates high-purity tomato juice EVs-like NPs, confirming murine alcoholic liver injury relief via type I interferon pathway [105]
Microfluidics/Acoustofluidics	Efficient separation of EVs using acoustic waves or microfluidic chips	Rapid (< 1 h), high purity (> 90%)^[107]^; suitable for trace samples [108]	High equipment requirements, low prevalence [109]; further validation of clinical applicability required [110]	This technology uses an integrated DLD chip to rapidly sort plant EVs from citrus extracts for real-time immunomodulatory screening [109]

For example, ultracentrifugation generally does not cause vesicle rupture; however, if rupture occurs, adjusting the centrifugation speed and duration can mitigate the damage. During ultrafiltration, the selection of a hollow fiber column with a larger surface area can reduce the number of concentration cycles, thereby minimizing the damage to the extracellular vesicles caused by prolonged shear forces.

### Impact of characterization methods on the assessment of PDEVs quality control

Characterization of extracellular vesicles serves as a crucial basis for evaluating their quality control status. In the characterisation of PDEVs, Multiple techniques complement each other in the characterization of PDEVs to enable comprehensive identification. For morphological observation, transmission electron microscopy (TEM) offers extremely high resolution, allowing for clear visualization of the ultrastructure of PDEVs and revealing their characteristic bilayer membrane and morphological features. However, vacuum operation and complex sample preparation procedures can damage the vesicle structure. In addition, TEM is expensive and requires advanced technical skills. Scanning electron microscopy (SEM) can directly display the surface morphology and three-dimensional structure of vesicles with relatively simple sample preparation; however, its resolution is insufficient for detailed visualization of small extracellular vesicles. Atomic force microscopy (AFM) enables non-destructive detection under near-physiological conditions and is highly sensitive to microscopic morphology. However, it suffers from complex operation procedures and a limited scanning range. Regarding particle size and concentration analysis, dynamic light scattering (DLS) is a simple and rapid technique suitable for monodisperse systems, but it is easily affected by large particles and cannot provide concentration information. Nanoparticle tracking analysis (NTA) can precisely track the movement trajectories of individual extracellular vesicles and simultaneously provide data on particle size and concentration, making it suitable for complex systems. However, NTA has strict requirements for sample quality. Nanoflow cytometry (NanoFCM) enables the multiparameter analysis of single particles with high sensitivity; however, it requires fluorescent labelling and involves high equipment and operation costs. In terms of molecular marker detection, western blotting can specifically identify marker proteins of extracellular vesicles, providing evidence at the molecular level; however, it is a time-consuming and labor-intensive procedure and is only applicable to known markers. In accordance with the "Expert Consensus on the Research and Application of Chinese Herbal Medicine Vesicles" and the recommendations of the International Society for Extracellular Vesicles (ISEV), techniques such as TEM, NTA, and western blotting have become essential methods for the identification of extracellular vesicles and related vesicles, ensuring the accuracy and reliability of PDEVs characterization (Table 3). Furthermore, single-vesicle flow cytometry or Raman spectroscopy can be used for high-resolution profiling of individual PDEVs. Advocating for the combination of proteomics, lipidomics, and metabolomics to establish comprehensive quality markers and functional annotations is also recommended.

**Table 3:** Current advantages and disadvantages of different characterization techniques for PDEVs

Technical name	Principle	Advantage		Disadvantage
NTA	By tracking the Brownian motion trajectories of each nanoparticle in the solution under a laser beam, its hydrodynamic diameter and concentration are calculated	1. Capable of measuring exosome particle size distribution (30–150 nm) and concentration, suitable for heterogeneous sample analysis [111] 2. Label-free, directly observes particle Brownian motion with a wide dynamic range (50–1000 nm) [112]		1. Inability to distinguish exosomes from other similarly-sized impurities (such as lipoproteins or protein aggregates) [113, 114] 2. High sample purity requirements, as impurities may interfere with counting accuracy [114]
DLS	By measuring the rate of laser scattered light intensity fluctuation caused by the Brownian motion of nanoparticles, the overall particle size distribution is analyzed	The rapid determination of average particle size and dispersion degree can be achieved with simple operation [115, 116]		Polydisperse samples exhibited interference, where contaminants (e.g., large microvesicles) masked exosomal signals^[112, 113]^. 2. Low resolution precluded discrimination between exosomes and non-vesicular particles[114]
TEM	High-energy electron beams penetrate ultra-thin samples and generate high-resolution 2D projection images based on differences in electron scattering and absorption across sample regions		High-resolution visualization of exosome morphology (such as cup-shaped structures) can be achieved, and immunogold labeling (such as CD63/CD9) can be combined to enhance specificity ^[111, 117]^. 2. The ability to distinguish exosomes from impurities such as apoptotic bodies [118]	1. Sample preparation procedures (e.g., fixation, dehydration) may compromise native structural integrity [111] 2. Quantification challenges were noted, coupled with high costs and prolonged processing time [115]
NanoFCM	Nanoparticles pass sequentially and individually through the laser detection zone under sheath flow, with multiparametric quantitative analysis via detecting their scattered light and fluorescence signals	High-throughput analysis of surface markers (e.g., CD9, CD81) enables discrimination of exosome subpopulations [119, 120] 2. Combined with fluorescent labeling, it enables detection of specific protein impurities (e.g., serum albumin) [121, 122]		1. Conventional flow cytometry exhibits a detection limit of ~ 300 nm, leading to frequent undersampling of small-sized exosomes [116, 122] 2. Antibody labeling is required, with potential cross-reactivity leading to false-positive results [120]
Western Blot	Proteins are separated by gel electrophoresis, transferred to a membrane, and specific proteins identified via target protein-specific antibody binding and chromogenic reactions	Exosomal markers (TSG101, Alix) and contaminants (ApoB lipoproteins) were confirmed [121] 2. Semi-quantitative analysis with high specificity was achieved [115]		1. Substantial sample input is required, with sensitivity dependent on antibody specificity [123] .2. The method fails to provide particle size distribution or quantitative concentration data [115]
Resistive Pulse Sensing (RPS)	Nanoparticles passing through a nanopore displace electrolyte, inducing transient resistance change across the pore with magnitude proportional to particle volume	High-resolution single-particle analysis enables discrimination of exosomes from impurities [112, 116] 2. Label-free direct measurement of particle size and zeta potential [111]		1. Sample purification prevented microchannel clogging [112, 115] 2. Low throughput limits applicability to mass screening [122]
Raman Spectroscopy	Chemical composition of samples is obtained by analyzing characteristic frequency-shifted light from laser-molecular bond inelastic scattering, bearing unique molecular vibrational info	Label-free profiling of exosomal biomolecules (e.g., nucleic acids, lipids) identified tumor-specific signatures [124] 2. Exosomes were effectively discriminated from functional microvesicles [125]		1. Weak signals necessitate enhancement techniques (e.g., SERS), though associated equipment incurs high costs [112] 2. Complex data analysis necessitates the establishment of standardized reference databases [123]
Mass Spectrometry	Sample molecules are first ionized, then separated by mass-to-charge ratio (m/z) in an electric/magnetic field, with molecular weight and structure determined via a detector	Comprehensive proteomic profiling of exosomes identified potential contaminants, including culture medium-derived proteins [126] 2. High sensitivity enabled detection of low-abundance biomarkers [113]		Sample pre-fractionation is required, involving costly instrumentation and complex data processing pipelines [126]

### Impact of storage conditions on PDEVs quality control and countermeasures

The storage conditions of plant-derived extracellular vesicles (PDEVs) play a pivotal role in quality control, significantly influencing vesicle structural integrity, bioactive molecule stability, and function maintenance. Studies have shown that factors such as storage temperature, storage duration, and buffer solution composition can markedly affect the physical and chemical properties of PDEVs.

For example, long-term storage at 4 °C or room temperature may lead to vesicle aggregation, membrane rupture, and nucleic acid and protein degradation [127]. Repeated freeze–thaw cycles at -80 °C can potentially disrupt the integrity of the lipid bilayer [128], and this disruption accelerates the loss of metabolites, proteins, and nucleic acids within PDEVs. Moreover, oxidative stress in the environment can expedite the inactivation of bioactive components within vesicles, such as miRNAs and antioxidant metabolites [15, 129], thereby diminishing their therapeutic potential, such as anti-inflammatory and anti-tumour effects [14, 54].

To address these challenges, the current quality control measures encompass multiple aspects of the production process. In terms of optimizing storage conditions, the addition of cryoprotectants, such as 1,3-butanediol, can significantly enhance the stability of vesicles at low temperatures [65], and an inert gas environment, such as nitrogen, can reduce oxidative damage [130]. For short-term storage, a temperature of 4 °C (≤ 7 days) is recommended, whereas for long-term preservation, a single freeze at -80 °C is required, and repeated freeze–thaw cycles should be avoided [52, 128]. Regarding standardized pretreatment, removing proteases and nucleases from the storage solution via ultrafiltration or size-exclusion chromatography can reduce the risk of degradation of biological macromolecules [10, 131], and coating vesicles with biocompatible materials, such as trehalose or polyvinyl alcohol, can enhance membrane stability [19, 132].

In the realm of dynamic monitoring technologies, the combination of nanoscale flow cytometry (nFCM) and surface plasmon resonance (SPR) enables real-time assessment of particle size distribution, surface markers, and cellular uptake efficiency of vesicles after storage [133, 134], and proteomic and transcriptomic analyses can verify the retention rate of functional molecules after storage [135, 136].

Finally, engineering modifications that involve enhancing the rigidity of the vesicle membrane through gene editing or chemical modification, such as overexpressing plant-specific sphingolipids or introducing cholesterol analogs, can improve the storage resistance of PDEVs [137, 138]. In the future, it is necessary to establish a unified storage standard across different species (such as ISO/TS 21387) and develop biomimetic preservation systems (such as plant matrix simulated solutions) to maintain the natural activity of PDEVs [139, 140]. These measures will contribute to achieving a higher level of quality control in the PDEVs.

## Future research directions

For the quality control of PDEVs, technological upgrades are required for four key aspects: plant raw materials, isolation and purification, characterization methods, and storage conditions. Integrating these four aspects into an organic whole is essential to achieve optimal quality control of PDEVs.

### Continuous upgrades and modifications of the TIBS

The Temporary Immersion Bioreactor System (TIBS) has demonstrated exceptional quality control capabilities within the Plant-Derived Extracellular Vesicles (PDEVs) quality control framework. Machine deep learning can continuously "domesticate" real experimental data to serve as a reference for the high-level quality control of standardized processes [141]. TIBS operational parameters, such as aeration time, intermittent immersion frequency, immersion duration, and degassing time, influence the quality control of PDEVs by affecting the quality of raw materials [142]. By establishing a deep learning model that integrates TIBS parameters, PDEVs characterization data, and multi-omics data to construct a multidimensional feature space, the TIBS system can rapidly and accurately identify conditions for producing PDEVs with high-quality control. This methodology has already achieved successful applications in microbial fermentation; for instance, random forest algorithms have been used to correlate fermentation temperature, carbon–nitrogen ratio, and product yield in Streptomyces colelicolor cultures, achieving a yield prediction accuracy of 89% [143]. For PDEVs-specific scenarios, Convolutional Neural Networks (CNNs) can be employed to process microscopic image data, achieving a subtype recognition rate of 87% [144]. Combining CNNs with Long Short-Term Memory (LSTM) networks to process time-series process data further aligns with the current frontier of "image-text fusion" in artificial intelligence for biological processes [145]. For example, fusing the dynamic numerical changes in the pH of the culture medium during TIBS operation with PDEVs characterization data enables rapid screening of the optimal pH for PDEVs under the best quality state, and facilitates intelligent control for subsequent scale-up to ensure quality control. This fusion allows for dynamic prediction of PDEVs quality attributes while avoiding over-speculation. When developing a digital twin system for TIBS, the focus should first be placed on a simplified, function-oriented virtual simulation platform rather than full-process reconstruction, which is consistent with the current status of digital twin applications in biomanufacturing [146, 147]. For instance, a digital twin model of E. coli fermentation was used to simulate the effects of agitation speed and dissolved oxygen on bacterial biomass, reducing experimental trial-and-error by 30% [148, 149]. Building on this precedent, a dedicated model for temporary immersion bioreactors can be initially constructed to simulate the impact of immersion frequency and duration on PDEVs yield and quality control. These models will first rely on offline experimental data for calibration, with real-time parameter optimization as a long-term goal—clearly distinguishing between "currently achievable simplified simulations" and "full-process digital twins in the future." The development of intelligent quality control traceability systems also has actionable precedents in the biopharmaceutical field. Graph Neural Networks (GNNs) have been applied in vaccine production to construct a "raw material-intermediate-finished product" quality association map [150], enabling rapid analysis of the root causes of purity deviations (e.g., identifying contaminated medium batches). Extending this framework to PDEVs, GNNs can be used to build a full-chain quality association map covering PDEVs sources, TIBS operational parameters, and final PDEVs characterization results.Synthetic biology-driven customized plant cell culture systems provide existing technical support for the engineering modification of PDEVs [151]. In Arabidopsis thaliana, the oleosin promoter can specifically drive lipid synthesis in seed cells; when applied to the regulation of PDEVs secretion, it has initially achieved an increase in vesicle yield in specific cell types [152, 153]. In terms of gene circuit design, the use of tissue-specific promoters to regulate the synthesis of target products in plant cells has been realized. The CRISPR-dCas9 system has become quite mature for the precise regulation of specific gene expression in plant cells [154]; for example, this system has been used to regulate small RNA production in rice [155], which provides direct technical reference for the precise regulation of nucleic acid components carried by PDEVs. In the reconstruction of metabolic pathways to optimize the bioactive components of PDEVs, modular engineering strategies have practical case support. For instance, in Arabidopsis thaliana, researchers improved the stability of cell membranes by modifying the sphingolipid metabolic network [156]; when applied to the optimization of PDEVs membrane structure, an initial improvement in the integrity of vesicles during storage has been observed. Membrane protein directional insertion technology has also been applied in the preparation of artificial vesicles; by optimizing the composition of the insertion buffer, the natural conformation and function of membrane proteins can be maintained [157]. These existing technical achievements provide a clear technical path for accurately improving the quality control of PDEVs. In the future, the cultivation advantages of TIBS can be further combined to realize the large-scale preparation and functional optimization of PDEVs.

### Multidisciplinary approaches for enhancing isolation and purification technologies

As emphasized in previous analyses, the isolation and purification of PDEVs remain challenging due to issues such as vesicle aggregation induced by traditional ultracentrifugation, low recovery rates, difficulties in scaling up, and the high costs and limited targeting capabilities of antibody-based capture techniques [13, 158–160]. Future technological breakthroughs require the in-depth integration of cutting-edge methods from multiple disciplines to establish efficient, standardized, and industrially scalable isolation and purification systems for these compounds.

#### Synergistic innovations in physics and materials science

The existing applications of microfluidic chip technology in the field of biological particle separation provide a technical foundation for the continuous sorting of PDEVs. Currently, microfluidic chips have been used for the size-based sorting of exosomes; by designing microchannels with specific pore sizes (50–200 nm) and combining dielectrophoresis technology, efficient separation of tumor-derived exosomes has been achieved [161, 162]. Drawing on this mature technology, microfluidic channels with specific pore sizes and surface chemical properties (e.g., hydrophilic coatings) can be designed according to the characteristics of PDEVs, and then coupled with dielectrophoresis or acoustic-assisted technology to realize continuous-flow sorting of PDEVs. In terms of acoustofluidic sorting, studies have utilized differences in acoustic impedance between different particles to successfully separate exosomes from protein impurities in blood, achieving a sorting purity of over 90% with a vesicle damage rate of less than 5% [163]. Adapting this technology to PDEVs isolation can lead to the development of a high-throughput, low-damage acoustofluidic sorting platform, addressing the aggregation issue of traditional centrifugation methods. In the field of nanoporous membrane filtration systems, Atomic Layer Deposition (ALD) technology enables precise regulation of membrane pore sizes [164]. Researchers have prepared alumina membranes on polymer substrates via ALD, controlling the pore size within the range of 30–200 nm, which has been successfully used for the retention and purification of yeast-derived vesicles. For PDEVs, this technology can be employed to prepare graphene or alumina membranes; surface charge modification (e.g., coating with the cationic polymer polyethyleneimine) can enhance the selective adsorption of negatively charged PDEVs [90]. In isolation experiments of garlic-derived PDEVs, the adsorption efficiency of cation-modified membranes was 40% higher than that of unmodified membranes, and they could effectively remove protein impurities. These technological and material advancements are expected to address the problems of easy aggregation and high damage rates of PDEVs in traditional isolation processes. Meanwhile, the modified membranes regulated by ALD improve isolation specificity and purity, which is expected to enhance quality control in the PDEVs isolation process from core dimensions such as isolation efficiency, vesicle integrity, and impurity removal efficacy.

#### Intelligent optimization through chemistry and computer science integration

The existing practice of machine learning in chromatographic condition optimization provides a basis for the development of hybrid chromatographic strategies for PDEVs. For example, in antibody purification processes, researchers have used machine learning to analyze the relationship between the amino acid composition of protein surfaces and chromatographic retention behavior, dynamically optimizing the pH and salt concentration of ion-exchange chromatography to improve product purity [164, 165]. Drawing on this idea, by analyzing the surface proteome of PDEVs (e.g., transmembrane protein abundance), machine learning algorithms can be used to construct a correlation model of "protein characteristics—chromatographic conditions—purification effect," thereby dynamically optimizing the operating parameters of anion/cation exchange chromatography. In the purification research of citrus-derived PDEVs, the content of surface transmembrane proteins (e.g., CD63 homologous proteins) has been initially detected to adjust chromatographic conditions for improving the recovery rate of target vesicles [166, 167], laying the foundation for the further application of this technology. The construction of real-time monitoring and closed-loop feedback systems can rely on existing analytical technologies. Fluorescent labeling technology has been used to track the isolation process of PDEVs [168]; for instance, fluorescent dyes are used to label phospholipids on the vesicle surface to observe their elution behavior in the chromatographic column in real time. Raman spectroscopy can quickly analyze the component purity of vesicles; in the purification of grape-derived PDEVs, researchers used Raman spectral characteristic peaks (e.g., C-H vibration peaks of lipids) to real-time determine impurity content, enabling immediate adjustment of the purification process [169]. Combining these two technologies with a closed-loop feedback system can construct an automated "detection—analysis—regulation" process, ensuring the accuracy of the PDEVs purification process. The application of molecular docking simulation in ligand screening is quite mature and can be used to design biomimetic magnetic bead capture systems for PDEVs. For example, in the study of interactions between plant lectins and carbohydrates, molecular docking was used to screen lectins that can specifically bind to citrus pectin [170], which were then used to modify magnetic beads to achieve efficient enrichment of citrus-derived PDEVs. In addition, in terms of parameter optimization, three-dimensional response surface models and genetic algorithms have been used to optimize centrifugation processes [52, 135]. In the isolation of dandelion-derived PDEVs, researchers constructed a response surface model of "centrifugal force—buffer ionic strength—recovery rate" and optimized parameters using genetic algorithms, increasing the vesicle recovery rate from 45 to 72% while maintaining the retention rate of active components at 90%. Computational Fluid Dynamics (CFD) has also been used to simulate the flow field distribution during centrifugation; in the isolation of stem cell-derived extracellular vesicles, optimizing the rotor geometry through CFD reduced the damage to vesicles caused by shear forces, improving vesicle integrity by 25%. These technologies can be directly adapted to the isolation and optimization process of PDEVs and have the potential to enhance the quality control of PDEVs [99, 171].

#### Interdisciplinary applications of biology and engineering

Metabolites in PDEVs play a role in stabilizing their structure and physiological activity to a certain extent. The existing application of CRISPR-Cas9 gene editing technology in the regulation of plant cell metabolism provides a feasible path for the recombinant modification of PDEVs. For example, in Arabidopsis thaliana, researchers successfully altered the secretion of extracellular vesicles by regulating vesicle biosynthesis-related genes (e.g., Rab GTPase genes) using CRISPR-Cas9 [172, 173]. Based on this, genetic engineering technology can be used to make recombinant PDEVs express affinity tags (e.g., His-tag) to simplify subsequent purification processes [174]. In hairy root culture systems, the stable production of GFP-tagged PDEVs has been realized; target vesicles can be rapidly enriched through affinity chromatography, with a purity of over 88%, indicating that this technology already has practical application value [175]. The in-situ isolation technology developed using the photosensitive properties of chloroplast membranes can rely on existing design experience of plant bioreactors. For instance, in tobacco cell culture, researchers activated photosensitive proteins on chloroplast membranes through blue light irradiation to trigger vesicle release [176]. Integrating this technology into plant bioreactors can construct an integrated module for "release—enrichment—purification" in a sterile environment, reducing the loss of PDEVs during the transfer process. This design has been initially verified in the large-scale production of microalgae vesicles, providing a new direction for the industrial isolation of PDEVs.

### Establishment of standardized characterization systems

To ensure the quality comparability of PDEVs from diverse sources, it is imperative to establish a unified evaluation criterion. This involves the preparation of reference materials where representative plant species, such as Vitis vinifera and Zingiber officinale, should be selected for standard sample preparation. The key parameters to be defined include characteristic marker proteins, such as PAT family proteins [1], fingerprint spectra, such as infrared spectral characteristic peaks [177], and bioactivity benchmarks, such as anti-inflammatory potency [54]. In addition, intelligent characterization platforms must be developed. Integrated detection systems should be developed to enable automated counting and particle size analysis via nanoflow cytometry [52], real-time monitoring of molecular interactions using surface plasmon resonance [72], and rapid identification of vesicle origins using Raman spectroscopy [9]. Moreover, stability-prediction models are required. Machine learning models should be constructed based on accelerated degradation test data to predict activity decay patterns under different storage conditions (lyophilized/liquid) [178], structural integrity changes during delivery [167], and the relationship between the in vivo circulation half-life and temperature [179]. Additionally, advanced characterization techniques such as correlative light-electron microscopy (CLEM) should be employed to systematically evaluate the impact of new methods on vesicle membrane integrity, cargo composition, and bioactivity [168].

### Enhancement of PDEVs storage technologies

The current storage of PDEVs predominantly relies on low-temperature conditions (e.g., 4 °C or − 80 °C), which entails challenges such as high energy consumption, transportation limitations, and vesicle integrity damage due to repeated freeze–thaw cycles [51, 180]. Although technologies such as nanohydrogen have shown promise in extending the storage duration, multi-technology collaborative innovation is required to overcome these bottlenecks. The research directions include the following.

#### Development and optimization of novel stabilizers

Existing studies indicate that PDEVs often experience functional decline during storage owing to oxidative stress and lipid peroxidation of the membrane [181]. Future research could focus on screening natural antioxidants (e.g., polyphenols and flavonoids) or synthetic polymers (e.g., polyethylene glycol modification) to enhance physicochemical stability by regulating vesicle surface charge or forming protective hydration layers [179, 182]. Additionally, inspiration from red blood cell preservation techniques can be used to develop specialized cryopreservation solutions tailored to plant vesicle membrane properties to minimize low-temperature-induced damage [51, 87]. Technologies such as nanohydrogen or inert gas may also be explored to prolong the shelf life of PDEVs.

#### Application of bioinspired modification technologies

Genetic engineering or chemical conjugation can introduce stabilizing domains, such as hydrophobin coatings or glycosylation modifications on vesicle surfaces, enhancing their tolerance to extreme conditions [5, 6]. For example, the co-incubation of plant vesicles with heat shock proteins (HSPs) or the knockout of labile membrane protein genes using CRISPR-Cas9 technology may significantly extend their half-life [13]. Designing bioinspired mineralized coatings, such as silica encapsulation, which mimic the long-term survival mechanisms of pathogen-derived vesicles, represents another promising avenue [167].

#### Construction of intelligent responsive storage systems

The combination of microfluidic technologies with environment-responsive materials, such as temperature- and pH-sensitive hydrogels, enables the development of dynamically regulated storage platforms. For example, encapsulating vesicles in sodium alginate-chitosan microcapsules allows ambient temperature stability through the controlled release of antioxidants and humidity regulation [12, 19]. The integration of IoT sensors can further enable real-time monitoring of the redox potential and vesicle integrity, ensuring precise quality control [52].

#### Synergistic integration of multimodal technologies

Given the complexity of storage challenges, single technologies are insufficient to address them. Future research should explore combined strategies such as "low temperature + nanohydrogen + photodynamic synergy." For example, superimposing a weak magnetic field on nanohydrogen treatment may inhibit vesicle aggregation and delay functional decline [135, 183]. AI-based stability prediction models can expedite the screening of optimal combinations of storage conditions by analyzing vesicle omics data (including lipidomics and proteomics) to establish a library of stability biomarkers [135]. These combinations can play a significant role in the quality control supervision process of PDEVs.

#### Establishment of standardized evaluation systems

The lack of standardized evaluation criteria for storage efficacy calls for the creation of a multidimensional assessment framework that includes physical parameters such as particle size distribution and zeta potential, bioactivity indicators like cargo retention rate and cellular uptake efficiency, and functional markers such as anti-inflammatory and targeting abilities [181, 184]. Employing microfluidic resistive pulse sensing (MRPS) in conjunction with nanoflow cytometry enables single-vesicle-level monitoring of subpopulation changes before and after storage [181]. Through interdisciplinary technological innovation and standardized system construction, breakthroughs in PDEVs storage technology are expected to facilitate their translation from laboratory research to clinical applications and industrialization.

## Conclusion and prospects

As nanoscale vesicles with a lipid bilayer structure secreted by plant cells, PDEVs possess a unique composition that enriches lipids, proteins, nucleic acids, and secondary metabolites such as flavonoids and terpenoids. They not only play a crucial role in plant physiological activities but also exhibit dual core values in the biomedical field. On one hand, they can serve as natural therapeutic agents, exerting direct pharmacological effects in anti-inflammation, anti-tumor, wound repair, gut microbiota regulation, and other aspects. On the other hand, they can act as novel nanodrug carriers. With low immunogenicity, good gastrointestinal stability, and targeted delivery capabilities, they overcome the defects of traditional synthetic nanocarriers, such as toxicity and low bioavailability. Furthermore, their targeting ability and efficacy can be further enhanced through engineering modifications like surface modification and drug loading. However, the functional exertion and industrial application of PDEVs are highly dependent on strict quality control. Currently, there are significant bottlenecks in all four core links covered by its quality control system:Firstly, in terms of raw material preparation, plant genetic background, abiotic factors, biotic factors, and traditional processing techniques can all cause fluctuations in raw material quality. Although plant tissue culture and TIBS can alleviate this problem through a standardized environment, large-scale and directional regulation technologies are not yet mature. Secondly, in the isolation and purification process, existing methods have their own limitations. The juice extraction method for raw material treatment is prone to producing artificial vesicles, the enzymatic hydrolysis method introduces impurities, and the apoplastic fluid extraction method has low yield. However, the ideal "non-destructive continuous collection" mode combining TIBS with liquid culture still requires further process optimization. Thirdly, in the characterization process, although the combined system of "TEM + NTA + Western Blot" can realize the basic characterization of morphology, particle size, and markers, the lack of unified quality markers and quantitative standards makes it difficult to compare the results of different studies. Finally, in the storage process, short-term storage at 4℃ and long-term storage at − 80℃ are prone to cause vesicle aggregation, membrane rupture, and degradation of contents. Repeated freeze–thaw cycles will aggravate the damage to the lipid bilayer. The existing stabilization strategies such as adding cryoprotectants and inert gas environment have limited effects, and there is a lack of unified storage standards across species and efficient dynamic monitoring technologies. Future research on PDEVs should take "standardization, high efficiency, and industrialization" as the core goals, and build a full-chain quality control system through the integration of multidisciplinary technologies:In terms of raw material quality control, promote the combination of TIBS and artificial intelligence to achieve intelligent regulation, and realize the customized production of PDEVs with the help of synthetic biology technologies. In terms of isolation and purification, integrate microfluidics, atomic layer deposition, machine learning, and other technologies to develop efficient sorting and purification methods, and endow PDEVs with affinity tags through gene editing to simplify the purification process. In terms of characterization, establish unified quality standards and reference materials, develop integrated intelligent characterization platforms, and promote advanced technologies to achieve full-dimensional accurate evaluation. In terms of storage, develop new stabilizers and bionic storage systems, construct intelligent responsive storage platforms, and formulate cross-species storage standards. At the same time, break through key industrialization technologies, strengthen multidisciplinary intersections and industry-university-research collaboration, and finally promote the quality control of PDEVs from laboratory research to application and industrialization, making them a core force of "green nanocarriers" in the biomedical field.

## Data Availability

No datasets were generated or analysed during the current study.
